# Supplementary material for: Serum Amyloid A as a Potential Biomarker in Inflammatory Bowel Diseases, Especially in Patients with Low C-Reactive Protein
Source: Int J Mol Sci. 2024 Jan 18;25(2):1177. doi: 10.3390/ijms25021177 (PMC10816523; doi:10.3390/ijms25021177)
Supplement: Supplementary file 1 [file ijms-25-01177-s001.zip › ijms-2787935-supplementary.pdf]

Table S1: Correlation analyses with SAA

Groups (n)	Spearman's coefficient of correlation*			P-Value		
**All cases (306)**						
FC	0.365			< 0.001		
CRP	0.623			< 0.001		
NLR	0.381			< 0.001		
**CD (182), UC (116), IBDU (8)**	**CD**	**UC**	**IBDU**	**CD**	**UC**	**IBDU**
HBI	**0.082**	-	-	**0.276**	-	-
SCCAI		0.300	-	-	0.001	-
FC	0.376	0.300	**0.700**	< 0.001	0.001	**0.053**
CRP	0.626	0.607	**0.669**	< 0.001	< 0.001	**0.070**
NLR	0.405	0.294	**0.599**	< 0.001	0.002	**0.117**
**Disease duration (93, 113, 100)**	**0 – 5 years**	**6 – 15 years**	**≥ 16 years**	**0 – 5 years**	**6 – 15 years**	**≥ 16 years**
FC	0.480	0.333	0.245	< 0.001	< 0.001	0.014
CRP	0.640	0.662	0.550	< 0.001	< 0.001	< 0.001
NLR	0.403	0.454	0.243	< 0.001	< 0.001	0.015
**Extraintestinal manifestation (102, 204)**	**Yes**	**No**		**Yes**	**No**	
FC	0.275	0.415		0.005	< 0.001	
CRP	0.612	0.630		< 0.001	< 0.001	
NLR	0.484	0.337		< 0.001	< 0.001	
**History of resecting surgery (85, 221)**	**Yes**	**No**		**Yes**	**No**	
FC	0.289	0.393		0.007	< 0.001	
CRP	0.529	0.659		< 0.001	< 0.001	
NLR	0.308	0.399		0.004	< 0.001	
**Biologic therapy (152, 154)**	**Yes**	**No**		**Yes**	**No**	
FC	0.461	0.255		< 0.001	0.001	
CRP	0.625	0.618		< 0.001	< 0.001	
NLR	0.417	0.277		< 0.001	< 0.001	

**The values in bold are not significant; CD Crohn's disease, UC Ulcerative colitis, IBDU Inflammatory bowel disease unclassified*

Table S2: Correlation analyses with SAA for the Montreal classification

Groups (n)	Spearman's coefficient of correlation*	P-Value
**CD: L1 = terminal ileal (68)**		
FC	0.287	0.018
CRP	0.549	< 0.001
NLR	0.335	0.005
**CD: L2 = colonic (21)**		
FC	0.572	0.007
CRP	0.630	0.002
NLR	**0.393**	0.078
**CD: L3 = ileocolonic (84)**		
FC	0.417	< 0.001
CRP	0.699	< 0.001

NLR	0.431	< 0.001
**CD: L4 = isolated upper disease (11)**		
FC	0.689	0.019
CRP	**0.511**	0.109
NLR	**0.540**	0.087
**CD: B1 = non-stricturing, non-penetrating (70)**		
FC	0.484	< 0.001
CRP	0.574	< 0.001
NLR	0.486	< 0.001
**CD: B2 = stricturing (64)**		
FC	0.255	0.042
CRP	0.675	< 0.001
NLR	**0.231**	0.067
**CD: B3 = penetrating (63)**		
FC	0.313	0.013
CRP	0.700	< 0.001
NLR	0.362	0.004
**UC: E1 = ulcerative proctitis (12)**		
FC	**0.090**	0.780
CRP	0.828	< 0.001
NLR	**0.414**	0.181
**UC: E2 = left-sided UC (44)**		
FC	**0.251**	0.100
CRP	0.628	< 0.001
NLR	0.319	0.035
**UC: E3 = extensive UC (64)**		
FC	0.424	< 0.001
CRP	0.590	< 0.001
NLR	**0.200**	0.123

**The values in bold are not significant; L Location, B Behavior, E Extent, CD Crohn's disease, UC Ulcerative colitis*
